# Supplementary material for: Estimating the Risk of Human Herpesvirus 6 and Cytomegalovirus Transmission to Ugandan Infants from Viral Shedding in Saliva by Household Contacts
Source: Viruses. 2020 Feb 3;12(2):171. doi: 10.3390/v12020171 (PMC7077293; doi:10.3390/v12020171)
Supplement: Supplementary file 1 [file viruses-12-00171-s001.pdf]

Table S1. Total observed and interpolated exposures by virus and exposure source.

Virus	Total exposure weeks	Exposure source	Total weeks observed	Total weeks interpolated (%)
HHV-6	684	Secondary Children	544	140 (20.5)
		Mother	646	38 (5.6)
CMV	819	Secondary Children	647	172 (21.0)
		Mother	767	52 (6.3)

Table S2. Spearmen correlations and p-values for exposure outcomes (percent positive swab and summarized viral loads (VL, DNA copies/mL)) between secondary children and mothers within a household.

Virus	Endpoint	Spearman correlation (95% CI)	P-value
HHV-6	Pct. Positive	0.30 (-0.06, 0.59)	0.095
	Mean VL	0.33 (-0.03, 0.61)	0.074
	Maximum VL	0.29 (-0.07, 0.59)	0.111
CMV	Pct. Positive	-0.04 (-0.40, 0.32)	0.818
	Mean VL	0.06 (-0.31, 0.41)	0.752
	Maximum VL	0.06 (-0.31, 0.41)	0.768

Table S3: Comparison of exposure outcomes (percent positive swab and summarized viral loads (VL, DNA copies/mL)) by exposure source (secondary children, mothers, and household sum) between households by infant transmission status. Significant differences ($p < 0.05$) are bolded.

Virus	Endpoint	Exposure source	Household transmission	No household transmission	Difference	P-value[2]
HHV-6	Pct. Positive	Secondary Children	100.00	94.50	5.50	**0.003**
		Mother	76.00	40.06	35.94	0.105
		Household Sum	100.00	98.91	1.09	**0.008**
	Mean VL[1]	Secondary Children	4.08	3.07	1.00	**0.007**
		Mother	2.02	1.05	0.97	0.102
		Household Sum	4.12	3.41	0.71	**0.006**
	Maximum VL[1]	Secondary Children	4.63	3.68	0.95	**0.020**
		Mother	3.34	3.00	0.34	0.147
		Household Sum	4.63	3.90	0.72	**0.016**
CMV	Pct. Positive	Secondary Children	100.00	94.05	5.95	0.230
		Mother	5.41	2.18	3.22	0.622
		Household Sum	100.00	95.30	4.70	0.249
	Mean VL[1]	Secondary Children	3.46	3.05	0.41	0.179
		Mother	0.13	0.05	0.08	0.684
		Household Sum	3.47	3.05	0.42	0.179
	Maximum VL[1]	Secondary Children	4.42	4.34	0.08	0.377
		Mother	1.11	2.49	-1.38	0.653
		Household Sum	4.43	4.34	0.09	0.400

[1]Viral load, DNA copies/mL.
[2]Exact Wilcoxon rank sum test

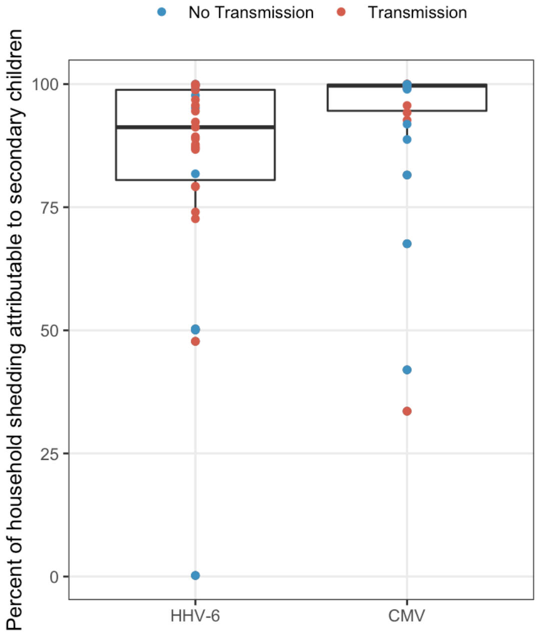

Figure S1. Composition of household sum viral load. Secondary household children comprise the majority of the summed household exposure measure compared to mothers. Percent of the weekly household sum exposure comprised by the secondary children viral load averaged over the exposure period. Boxes represent interquartile range; stems represent values within 1.5 of the IQR.

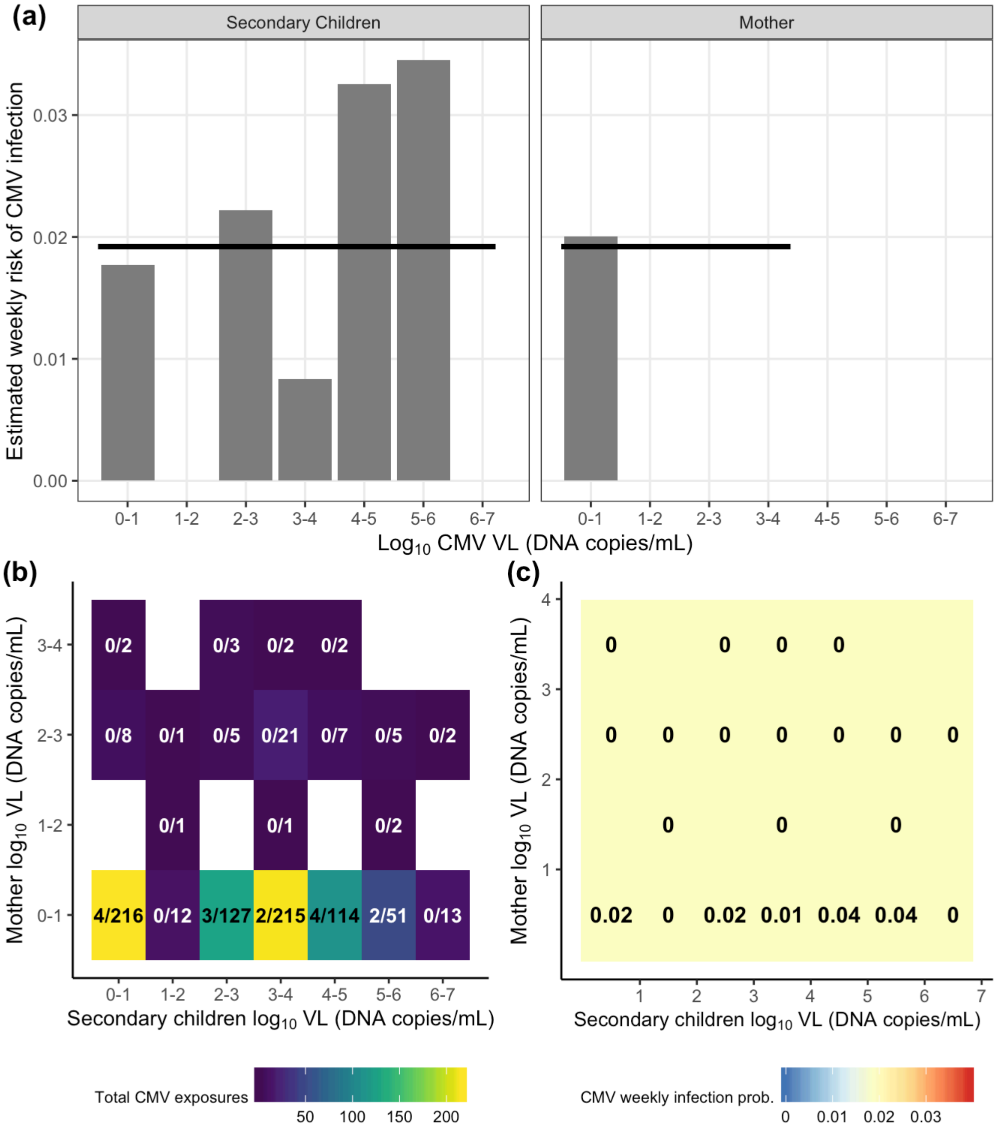

Figure S2: Estimation of CMV acquisition risk from exposures by week using the combined exposure model (Tables 2 and B1). (**a**) Individual CMV dose-response relationship for mother and secondary children oral viral load (VL) exposures where bars depict the percentage of infections observed among total exposures in the data and lines depict estimated risk from the model. (**b**) Heatmap depicting distribution of total combined exposures in a given viral load bin from mothers and secondary children in a household. Text depicts fraction of total infections over total exposures. (**c**) Heatmap depicting risk of CMV infection from combined household mother and secondary children exposures estimated from the model. Text depicts proportion of infections observed in the data using binned viral loads from (b) (equivalent to fraction displayed).
